# A Randomized, Double-Blind, Placebo-Controlled, Multicentre Trial of the Effects of a Shrimp Protein Hydrolysate on Blood Pressure

**DOI:** 10.1155/2019/2345042

**Published:** 2019-08-05

**Authors:** Kathy Musa-Veloso, Lina Paulionis, Tetyana Pelipyagina, Mal Evans

**Affiliations:** ^1^Food & Nutrition Group, Intertek Scientific & Regulatory Consultancy, 2233 Argentia Road, Suite 201, Mississauga, ON, Canada L5N 2X7; ^2^KGK Science Inc., 255 Queens Avenue, Suite 1440, London, ON, Canada N6A 5R8

## Abstract

In this randomized, double-blind, placebo-controlled, multicentre, parallel, 8-week study, the efficacy of a daily dose of 1200 mg of protein hydrolysate from Coldwater Shrimp (*Pandalus borealis*) on ambulatory and office blood pressure was investigated in 144 free-living adults with mild to moderate hypertension. The primary outcomes of the study were daytime ambulatory systolic blood pressure and office blood pressure. During the 8-week intervention period and in the intention-to-treat analysis (n=144), there were significant reductions in the group consuming the shrimp-derived protein hydrolysate relative to the placebo group in daytime ambulatory systolic blood pressure at 4 weeks (p=0.014) and at 8 weeks (p=0.002), and in office systolic blood pressure at 2 weeks (p=0.031) and 4 weeks (p=0.010), with a trend toward significance at 8 weeks (p=0.087). By 8 weeks, significant and favourable improvements in the group consuming the shrimp-derived protein hydrolysate relative to the placebo group were also observed for several secondary outcomes, including 24-hour ambulatory systolic and diastolic blood pressure, daytime ambulatory diastolic blood pressure, and daytime and 24-hour ambulatory mean arterial pressure. Also by Week 8, there was a statistically significant difference between groups in the distribution of subjects across National Institutes of Health-defined blood pressure categories (i.e., Normotensive, Prehypertensive, Stage 1 hypertension, and Stage 2 hypertension), with a more favourable distribution in the shrimp-derived protein hydrolysate group than in the placebo group (p=0.006). Based on exploratory analyses conducted only in participants in the shrimp-derived protein hydrolysate group, angiotensin II levels were significantly reduced relative to baseline. This study is registered at ClinicalTrials.gov NCT01974570.

## 1. Introduction

According to 2008 data collected by the World Health Organization (WHO) on noncommunicable diseases, globally, the overall prevalence of elevated blood pressure (BP) (defined as a systolic blood pressure [SBP] ≥140 mmHg or a diastolic blood pressure [DBP] ≥90 mmHg) in adults 25 years of age and older is 40% [1, 2]. Elevated BP, if left untreated, can lead to cardiovascular diseases (CVDs) (e.g., stroke, myocardial infarction, cardiac failure, congestive heart failure, and atrial fibrillation), renal disease and failure, cognitive decline (e.g., vascular dementia), and blindness [3, 4]. Worldwide, elevated blood pressure is estimated to cause 7.5 million deaths, which is about 12.8% of all deaths [2]. Thus, as a global target to be achieved by 2025, the WHO has called for a 25% relative reduction in the prevalence of raised BP or the containment of the prevalence of raised BP, according to national circumstances [5]. In order to achieve this target, dietary and lifestyle behaviours that effectively reduce BP levels (e.g., achieving a healthy body weight, avoiding tobacco use, drinking alcohol in moderation, increasing physical activity levels, and following a low-sodium diet rich in fruits and vegetables) must be adopted. With the worldwide surge in the aging population, however, the prevalence of elevated BP is steadily increasing, and additional interventions that could assist with the maintenance of normal BP levels are needed.

Marine-derived protein hydrolysates may be useful as adjunctive treatments in the management of hypertension or in the maintenance of normal BP levels. Using the spontaneously hypertensive rat as a model of hypertension, several different fish protein hydrolysates were demonstrated to have antihypertensive effects. These protein hydrolysates were derived from the bowels of Skipjack tuna (*Katsuwonus pelamis*), muscle of Bigeye tuna (*Thunnus obesus*), muscle of sardine (*Sardinops melanostictus*), loach (*Misgurnus anguillicaudatus*), head of cobia (*Rachycentron canadum*), backbone of ribbonfish (*Trichiurus haumela*), and skin of skate (*Okamejei kenojei*) [6–15]. In another study, however, protein hydrolysates of wild Atlantic cod (*Gadus morhua L*.), haddock (*Melanogrammus aeglefinus L*.), or farmed Atlantic salmon (*Salmo salar L*.) were found to be ineffective in reducing BP in spontaneously hypertensive rats [16]. While the reasons for the apparent ineffectiveness are not entirely clear, it is likely that the source of the protein (fish species and anatomical origin), the hydrolysing enzyme(s), the enzyme to substrate ratio, and the protein hydrolysis conditions (i.e., temperature, time, and pH) all could impact the presence of other potential bioactives, such as secretagogues, calciotropic hormones, and growth factors, as well as the resulting amino acid sequences [17].

Studies of the potential antihypertensive effects of protein hydrolysates derived from crustaceans are limited. In some studies, the in vitro inhibition of angiotensin-converting enzyme (ACE) was greater with shrimp protein hydrolysates than with fish protein hydrolysates [18, 19]. Nii et al. [20] previously demonstrated that the oral administration of an izumi shrimp (*Plesionika izumiae* Omori) hydrolysate significantly inhibited the age-associated spontaneous increase in BP in stroke-prone spontaneously hypertensive rats. The group went on to isolate two ACE inhibitory peptides from the izumi shrimp hydrolysate; their amino acid sequences were determined to be valine-tryptophan-tyrosine-histidine-threonine and valine-tryptophan [21]. The BP in stroke-prone spontaneously hypertensive rats was shown to decrease significantly after just a single oral administration of synthetic versions of the aforementioned two amino acid sequences [21]. Likewise, Gildberg et al. [22] reported high ACE inhibitory activity of a desalted protein hydrolysate from Northern shrimp (*Pandalus borealis*), with two novel ACE inhibitory tripeptides detected in the hydrolysate, namely, phenylalanine-threonine-tyrosine and phenylalanine-serine-tyrosine. Further, significant improvements in BP were observed when spontaneously hypertensive rats were administered 60 mg of the shrimp protein hydrolysate per kg body weight per day [22].

Clinical studies of the efficacy of hydrolysates derived from shrimp in reducing BP have not yet been conducted. In this study, we report the results of a randomized, double-blind, placebo-controlled, multicentre, parallel study in which the primary objectives were to assess the efficacy of desalted shrimp protein hydrolysate from Coldwater Shrimp (*Pandalus borealis*) on the changes from baseline in daytime ambulatory SBP and office SBP in individuals with mild to moderate hypertension.

## 2. Methods

### 2.1. Study Objectives and Design

The primary objectives of the study were to assess the effects of a shrimp-derived protein hydrolysate [hereinafter also referred to as a Refined Peptide Concentrate (RPC)] versus placebo on the changes from baseline over 8 weeks in daytime ambulatory SBP and office SBP. Secondary objectives were to assess the effects of the RPC versus placebo on 24-hour and night-time ambulatory SBP; 24-hour, daytime, night-time ambulatory and office DBP; and other endpoints (e.g., heart rate, fasting serum glucose and serum lipids, serum C-reactive protein, dietary variables from food records, urinary sodium). Exploratory objectives were to assess the effects of the RPC on blood levels of angiotensin I and II, aldosterone, renin, ACE activity, and low-density lipoprotein (LDL) oxidation.

The study was multicentre (21 centres/sites), randomized, double-blind (investigator, participants, and other site personnel all blinded), placebo-controlled, and parallel (two arms). The study was conducted from January 2014 to September 2015. Initially, twelve sites in North America (eleven sites in Canada and one site in the United States), and nine sites in Europe (three sites in Germany and six sites in the Czech Republic) were involved in the study; however, two sites in North America (both in Canada) were not successful in enrolling patients. The study was performed in accordance with the ethical principles that have their origins in the Declaration of Helsinki and its subsequent amendments, and in accordance with the International Council for Harmonisation of Technical Requirements for Registration of Pharmaceuticals for Human Use [23], and applicable regulatory requirements. This study is registered at ClinicalTrials.gov (ClinicalTrials.gov identifier: NCT01974570) [24].

### 2.2. Study Population

Individuals were recruited via direct e-mails, as well as via online and posted paper advertisements. Individuals had to meet all of the inclusion criteria and none of the exclusion criteria to qualify for the study.

The inclusion criteria for subject selection were as follows: (1) male or female aged 30 to 75 years, inclusive (independent and home-living); (2) if female, not of child-bearing potential or having a negative urine pregnancy test result and agreeing to use a medically approved method of birth control; (3) mild or moderate hypertension (SBP 140 to 160 mmHg and DBP ≤100 mmHg; mean of office BP measurements from three occasions used, i.e., the first two study visits during the run-in period and the baseline measurement); (4) body weight ≥60 kg; (5) stable body weight (self-reported weight gain or loss <5 kg in the past 3 months); (6) voluntary, written, and informed consent to participate in the study; and (7) agreement to comply with study procedures; to fast (at least 12 hours) and abstain from alcohol (2 days) prior to blood sampling; to abstain from alcohol (2 days), coffee (14 hours), and physical exercise (4 hours) prior to blood pressure measurement; and to abstain from donating blood during and for 30 days after the study. The exclusion criteria for subject selection were as follows: (1) females who were pregnant, breastfeeding, or planning to become pregnant during the course of the trial; (2) body mass index (BMI) ≥35 kg/m^2^; (3) antihypertensive drug treatment, regular high-dose nonsteroidal anti-inflammatory drug treatment, or use of cyclosporine or tacrolimusin; (4) Any history of CVD, dementia/cognitive impairments, hypertensive retinopathy, left ventricular dysfunction or peripheral artery disease, secondary hypertension, diabetes (Types 1 and 2), cancer within the past 5 years (excluding basal cell carcinoma), or any other disease or condition which, in the Investigator's opinion, could interfere with the results of the study or the safety of the subject; (5) clinically significant biochemistry, haematology, and/or urinalysis, at the Investigator's discretion; (6) dietary restriction (fish and other seafood allergies, citrus allergies, and multiple food allergies); (7) alcohol abuse [defined as the consumption of more than 14 portions of alcohol per week (one portion = 1 oz. spirits or 4 oz. wine or 11oz. medium strength beer / cider)] and illicit drug use, including smokers and tobacco/snuff/nicotine users; (8) use of natural health products intended for BP lowering within 30 days before randomization; and (9) participation in another clinical research trial within 30 days prior to randomization.

Each subject was allocated a randomization number according to a randomization scheme generated by www.randomization.com. A staff member not involved in any study procedure bottled and labelled the study product; labels were applied according to the randomization list. The investigator, study personnel (involved in product dispensing, visit assessments, conduct of the study, monitoring, and analysis), and the participants did not know what treatment had been assigned. Sealed individual randomization envelopes containing the randomization number and associated treatment were prepared and kept at the coordinating centre and as such, there was allocation concealment.

### 2.3. Description of Investigational Products

Study participants received either the shrimp-derived RPC or placebo tablets. Study participants were instructed to consume two 850 mg tablets of the shrimp-derived RPC or placebo, once daily, with water, before noon (and between meals). Each 850 mg tablet of active test product contained 600 mg of protein hydrolysate from desalted Coldwater Shrimp (*Pandalus borealis*); thus, those in the RPC group received 1200 mg of protein hydrolysate from desalted Coldwater Shrimp daily.

The active and placebo products were identical in composition, except that the active product contained (per tablet) 600 mg of protein hydrolysate from Coldwater Shrimp (*Pandalus borealis*), while the placebo product contained (per tablet) 10 mg of Rainbow trout fish oil (used to match the taste and smell of the investigational products) and a higher amount of microcrystalline cellulose. There were no differences in the taste, smell, colour, size, texture, or packaging between the active test product and placebo; thus, both products were matched in taste, smell, and appearance.

Compliance with the intake of the tablets was assessed by counting the returned product at each visit. Compliance was calculated as:

(1) $$\begin{array}{c} \left(\;\frac{\#\text{ of tablets consumed}}{\#\text{ of tablets expected to be consumed}}\;\right)x\;\;100\% \end{array}$$

### 2.4. Assessments

#### 2.4.1. Office BP and Office Heart Rate

Office SBP and DBP and office heart rate were measured at screening (-4 weeks), run-in (-2 weeks), baseline (Week 0), Week 2, Week 4 (midpoint of study), and Week 8 (end of study). Office BP was measured according to Dieterle [25] and Pickering et al. [26]. Subjects were in a seated position with their legs uncrossed, feet flat on the floor, and backs comfortably flush against the back of a chair for 5 minutes prior to and for the entire period during the BP measurements. Using a random-zero mercury or digital sphygmomanometer, trained personnel measured BP. BP was initially measured in both arms and the arm with the higher value was used for all subsequent BP measurements; the arm was supported at heart level. At each office visit, three BP measurements were taken over 2-minute intervals with the first measurement discarded and the latter two measurements averaged. Study eligibility was determined by averaging the BP measurements taken over three office visits (screening [Week -4], run-in [Week -2], and baseline [Week 0]). The same recording method and the same equipment were used for each subject throughout the study.

Office heart rate was measured by radial arterial measurement and counting of arterial pulses per minute. At each visit, the number of arterial pulses per minute was measured three times and the three measurements were averaged to represent the participant's heart rate during the visit.

#### 2.4.2. Ambulatory BP and Ambulatory Heart Rate

Ambulatory SBP, DBP, and heart rate were measured at baseline, Week 4, and Week 8 using a 24-hour Ambulatory Blood Pressure (ABP) monitor (Spacelabs Medical, model number 90807-1Q), according to Dieterle [25] and O'Brien et al. [27]. Subjects were fitted with an ambulatory BP monitor, with the cuff secured on the nondominant arm for at least 25 consecutive hours. All ambulatory BP monitors and cuffs were coded, and each subject was fitted with the same monitor and cuff for each wearing occasion. Ambulatory measurements were programmed to occur at 15-minute intervals from 7:00 AM to 10:00 PM inclusive and at 20-minute intervals from 10:00 PM to 7:00 AM inclusive. Daytime ambulatory BP and daytime ambulatory heart rate were defined as 8:00 AM to 8:00 PM inclusive, and night-time ambulatory BP and night-time ambulatory heart rate were defined as 12:00 AM to 6:00 AM inclusive, similar to how “daytime” and “night-time” were defined in other ambulatory BP studies [28–35]. The selection of these time ranges for “daytime” and “night-time” resulted in the elimination, from the daytime and night-time measures, values collected from 6:00 AM to 8:00 AM and from 8:00 PM to 12:00 AM, during which subjects would have been awakening or falling asleep, respectively, and during which there would be considerable variations in blood pressure.

#### 2.4.3. Height, Weight, BMI

Height was measured at screening and weight was measured at screening, run-in, baseline, and Weeks 2, 4, and 8. The Health O Meter Professional Scale was used to measure height (reported in centimetres) and weight (reported in kilograms). Measurement of height was performed with shoes removed, knees straight, and head held upright. Measurement of weight was performed with the subjects in light clothing, shoes removed, and bladder empty. Subjects were weighed on the same scale at all visits. At least two separate body weight measurements were taken at each visit. If the two measurements were more than 0.5 kg (1.1 lbs) apart, a third measurement was taken, and the two closest values were selected and averaged. BMI was calculated at screening, run-in, baseline, and Weeks 2, 4, and 8.

#### 2.4.4. Other

Three-day food records (two weekdays and one weekend day) were completed in the week prior to the baseline, Week 4, and Week 8 visits. Adverse events (AEs) were assessed at Weeks 2, 4, and 8.

### 2.5. Laboratory Analysis

Collection of blood (subjects were fasted ≥12 hours) for the laboratory analysis of lipids (total cholesterol, high-density lipoprotein- (HDL-) cholesterol, LDL-cholesterol, triglycerides), C-reactive protein, angiotensin I and II, aldosterone, renin, ACE activity and LDL oxidation occurred at baseline and Week 8. A 24-hour collection of urine, for the measurement of urine sodium, urine creatinine, and urine volume also occurred at baseline and Week 8. Blood collection (subjects were fasted ≥12 hours) for safety endpoints (complete blood count, glucose, creatinine, estimated glomerular filtration rate, sodium, potassium, chloride, aspartate aminotransferase, alanine aminotransferase, gamma glutamyltransferase, and bilirubin) was conducted at screening and Week 8. It should be noted that the assessment of angiotensin I and II, aldosterone, renin, ACE activity, and LDL oxidation was conducted using the baseline and Week 8 blood samples only for subjects who were randomized to the RPC group, to explore the potential mechanism of effect.

Subjects' blood samples were stored at -40°C or, if the site did not have this capability, at -20°C, for a period not exceeding 14 days after blood collection. All the clinical research sites within a particular country used the same laboratory for the analysis of blood and urine (with the exception of urine pregnancy tests, which were done at each clinic). Quality assurance and clinical ranges used (for the laboratory analysis) were in accordance with each respective laboratory's guidelines.

Just prior to analysis, blood samples intended for enzyme-linked immunosorbent assay (ELISA) analysis were kept at temperatures of between 2 and 8°C (aldosterone, ACE activity, renin) or at 4°C (angiotensin I, angiotensin II). ELISA kits were used for their analysis (catalogue numbers: KGE016, DACE00, DREN00, ab136934, ab108796, respectively).

### 2.6. Statistical Analysis

Based on a previous unpublished study, a standard deviation (SD) of 7 mmHg was used for the mean change in daytime ambulatory SBP and a SD of 12 mmHg was used for the mean change in office SBP. With an anticipated attrition rate of 12% and in order to detect a difference of 4 or 6 mmHg in the mean change in daytime ambulatory SBP or office SBP, respectively, after an 8-week supplementation period with a probability of 80% at alpha level 0.05, the required sample size was estimated as 55 or 72 randomized subjects per group, respectively. Therefore, the sample size was determined to be 144 subjects (72 subjects per group), randomized in a 1:1 ratio to one of two groups.

Two populations were used for the efficacy analysis: (i) the intention-to-treat (ITT) population which consisted of all participants who received either product, and on whom any post-randomization efficacy information was available; and (ii) the per-protocol (PP) population, which consisted of all participants who consumed at least 80% of either product, did not have any major protocol violations and completed all study visits and procedures connected with the measurement of the primary variables. The safety analysis was conducted on the safety population, which consisted of all participants who received any amount of either product and on whom any postrandomization safety information was available. All missing values in the ITT analysis were imputed with the most recent previously available value (“last-observation-carried-forward” or LOCF imputation). No imputation was performed for missing values of safety variables.

Numerical efficacy endpoints were tested for significance between groups by analysis of covariance (ANCOVA). The dependent variable was the value at each visit, the factor was the treatment group, and the value at baseline (Week 0) was the covariate. For parameters that required a transformation, the transformed values were used in the ANCOVA model. Numerical endpoints that were intractably nonnormal were assessed by the Mann-Whitney U test. A within-group analysis on numeric endpoints was done using the Student's paired t-test or, in the case of intractable nonnormality, the Wilcoxon sign rank test.

For numeric safety endpoints, the data were presented and analysed using the same methods as the efficacy data. For AEs, a descriptive analysis was provided by body system and treatment group; also, the nature, incidence, severity, and causality were reported for each AE.

Probabilities ≤0.05 were considered statistically significant. All statistical analyses were completed using the R Statistical Software Package Version 3.2.2 for Microsoft Windows [36].

## 3. Results

**Figure 1:**
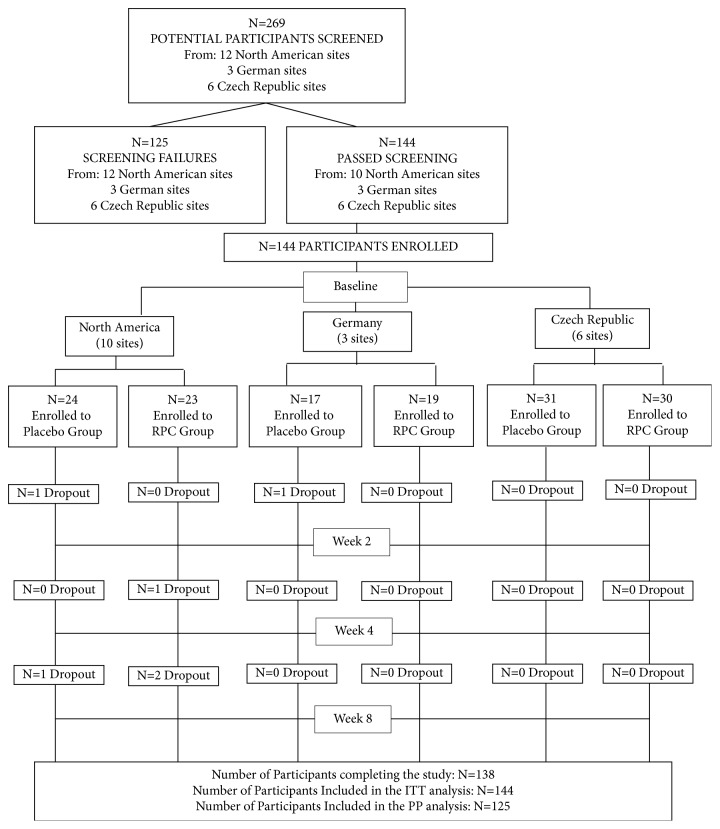
Flowchart of study participants.

As outlined in Figure 1, a total of 269 individuals were screened for potential inclusion into the study with 144 enrolled into the study (72 in each group). Efficacy and safety data were available for all 144 subjects; thus, the ITT and safety analyses included data from all 144 subjects. A total of 138 subjects completed the study (69 in each group). Six subjects (three in each group) dropped out from the study for personal reasons. Of the 138 completers, eight were incorrectly enrolled into the study based on baseline BP and/or thyroid stimulating hormone level, fasting glucose level, or smoking status, and for an additional five subjects, ambulatory BP data were not available. These subjects were excluded from the PP population which, as a result, consisted of 125 subjects (62 in the placebo group and 63 in the RPC group).

Subjects in both the RPC and placebo groups were generally well-matched, with no significant differences between groups in the majority of the demographic (Table 1) and baseline (Table 2) variables assessed, except for SBP, which was slightly but significantly greater in the placebo group compared to the RPC group. Average compliance with intake of the tablets over the 8 weeks was high (≥97.5%) for both groups, with no statistically significant differences observed between groups in either population (data not shown).

The effects of RPC versus placebo on ambulatory BP and office BP in the ITT population are summarized in Tables 3 and 4, respectively. Daytime ambulatory SBP was significantly reduced from baseline in the RPC group relative to the placebo group, both at 4 weeks (p=0.014) and at 8 weeks (p=0.002). Office SBP was significantly reduced in the RPC group relative to the placebo group at 2 weeks (p=0.031) and 4 weeks (p=0.010), with a trend towards significance at 8 weeks (p=0.087). Similar to daytime ambulatory SBP, 24-hour ambulatory SBP was also significantly reduced from baseline in the RPC group relative to the placebo group at both 4 weeks (p=0.015) and 8 weeks (p=0.006), while night-time ambulatory SBP was significantly reduced in the RPC group relative to the placebo group at 4 weeks (p=0.007) but not 8 weeks (p=0.166). Although changes from baseline in night-time ambulatory DBP were not significantly different between groups at any of the time points assessed, daytime ambulatory DBP was significantly reduced in the RPC group versus the placebo group, both at 4 weeks (p=0.036) and at 8 weeks (p=0.004), while at 8 weeks (but not 4 weeks), 24-hour ambulatory DBP was significantly reduced in the RPC group relative to the placebo group (p=0.047). There were no significant differences between the RPC and placebo groups in office DBP at any of the time points assessed. Daytime and 24-hour ambulatory mean arterial pressure were significantly reduced from baseline in the RPC group relative to the placebo group at 4 and 8 weeks; a between-group significant change in night-time mean arterial pressure, favouring RPC over placebo, was observed only at 4 weeks (data not shown).

**Figure 2:**
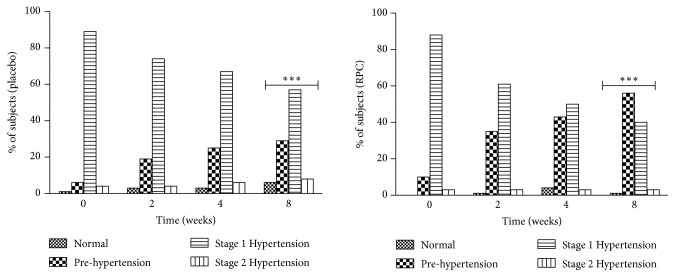
Proportion of ITT subjects in NIH-defined blood pressure categories at weeks 0, 2, 4, and 8 of the study based on office blood pressure. ITT: intention-to-treat; NIH: National Institutes of Health; RPC: Refined Peptide Concentrate; ∗∗∗: p=0.006, for the difference between groups in the distribution of subjects across NIH-defined blood pressure categories, favouring RPC over placebo.

Taking into consideration the changes in the distribution of subjects across National Institutes of Health- (NIH-) defined blood pressure categories over the course of the study (Figure 2), in both groups, based on office SBP and/or DBP levels, there was a favourable increase in the proportion of subjects classified as “Normal” or “Prehypertensive” from baseline to 8 weeks. In the placebo group, the percentage of subjects classified as “Normal” or “Prehypertensive” was 7% (5/72) at baseline compared to 35% (25/72) at 8 weeks. In the RPC group, the percentage of subjects classified as “Normal” or “Prehypertensive” was 10% (7/72) at baseline compared to 57% (41/72) at 8 weeks. Although there was a placebo effect, which is a well-characterized phenomenon in hypertension studies, consumption of RPC versus placebo resulted in the shifting of a greater proportion of subjects from hypertensive categories (i.e., Stage 1 or Stage 2 hypertension) into Normal/Prehypertensive categories. At Week 8, there was a statistically significant difference between groups in the distribution of subjects across NIH-defined blood pressure categories, favouring RPC over placebo (p=0.006). By Week 8, the proportion of participants in the placebo and RPC groups who were categorized as having Normal blood pressure, Prehypertension, Stage 1 hypertension, and Stage 2 hypertension was 6% versus 1%, 29% versus 56%, 57% versus 40%, and 8% versus 3%, respectively.

Daytime, night-time, and 24-hour ambulatory heart rate, office heart rate, C-reactive protein, urine sodium, and blood lipids (triglycerides, total cholesterol, HDL cholesterol, LDL cholesterol, non-HDL cholesterol, total cholesterol:HDL cholesterol), measured at Weeks 2, 4, and/or 8, remained similar to baseline values, and there were no statistically significant differences between groups in the changes in these outcomes (data not shown). Results from 3-day food records conducted in the week prior to the baseline, Week 4, and Week 8 visits showed no statistically significant differences between groups in the average daily intake of energy (calories) and the percent contribution of protein, carbohydrate, and fat intake to total daily energy intake (data not shown). In the RPC group, angiotensin II levels were significantly reduced from baseline to Week 8 (-0.026 ± 0.060 pg/mL; p<0.001); the remaining exploratory variables (oxidized LDL, ACE activity, renin, angiotensin I, and aldosterone) did not statistically significantly change during the intervention period (Table 5).

For all variables and outcomes assessed, the PP population analyses were generally similar (in direction and statistical significance) to those of the ITT population analyses.

A total of 70 AEs (28 in placebo group and 42 in RPC group) were reported during the study (56 mild, 13 moderate, and one severe in intensity). One AE (in RPC group) was determined as having the ‘most probable' relationship to treatment, ten AEs (six in placebo group and four in RPC group) were determined as having a ‘possible' relationship to treatment, 15 AEs (five in placebo group and ten in RPC group) were determined to have an ‘unlikely' relationship to treatment, and 44 AEs (17 in placebo group and 27 in RPC group) were determined to be ‘unrelated' to treatment. The one AE that was rated as ‘severe' in intensity (accelerated hypertension) occurred in the placebo group and was determined to be unrelated to treatment. The one AE (nausea) that was determined as having the ‘most probable' relationship to treatment occurred in the RPC group and was rated as moderate in intensity. The ten AEs determined to be ‘possibly' related to treatment were RPC group—euphoric mood (n=1), fatigue (n=1), upper abdominal pain (n=1), headache (n=1); placebo group—conjunctival haemorrhage (n=1), upper abdominal pain (n=1), dermatitis (n=2), diarrhoea (n=2); all ten AEs were rated as mild in intensity. There were no serious AEs reported in the study. Changes from baseline to Week 8 in haematological and clinical chemistry parameters, urine safety parameters (urine creatinine concentration, urine volume, and urine creatinine amount), and anthropometric variables such as body weight and BMI were not significantly different between groups.

## 4. Discussion

Elevated BP or hypertension is one of the key independent risk factors for CVD, a global health issue that is estimated to affect ~20% to 30% of the world's adult population [37, 38]. Clinically, hypertension is characterized as having SBP above 140 mmHg and/or DBP above 90 mmHg, and/or the current use of any antihypertensive medication [26]. High BP is strongly correlated with mortality, highlighting the need for therapeutic approaches to reduce BP and reduce CVD risk.

ACE converts angiotensin I to angiotensin II (which is a potent vasoconstrictor), inhibits the activity of the vasodilator bradykinin, and increases aldosterone secretion from the adrenal cortex, which has a tendency to cause elevated BP by modulating renal sodium and water retention [39]. ACE inhibition is therefore a target in the clinical management of elevated BP.

It was suggested by Cheung et al. [40] that the amino acid residues at the COOH- and NH_2_- terminals are important determinants of the ACE inhibitory potency of a fish protein hydrolysate. Specifically, when glycine was at the NH_2_-terminal, the COOH- residues that most effectively inhibited ACE were tryptophan, tyrosine, or proline. When glycine was at the COOH-terminal, the NH_2_ residues that most effectively inhibited ACE were valine, isoleucine, and arginine. In a fish protein hydrolysate derived from sardine muscle, the valine-tyrosine hydrolysate is considered to have the strongest ACE inhibitory effect, a finding aligned with that of Cheung et al. [40], who reported that valine-tyrosine ranked in the top ten of a total of 51 dipeptides in terms of having the most potent inhibition of ACE. In a protein hydrolysate of pink salmon (*Oncorhynchus gorbuscha*), it was demonstrated, in vitro, that ten dipeptides and ten tripeptides had ACE inhibitory activities, and that all 20 peptides had aliphatic (i.e., glycine, alanine, valine, leucine, and isoleucine) and aromatic (phenylalanine, tryptophan, tyrosine, and histidine) amino acids in their sequence [21]. The RPC administered in our study was derived from Coldwater Shrimp (*Pandalus borealis*). The ACE inhibitory tripeptides that have been detected in our hydrolysate from Northern shrimp (*Pandalus borealis*) include phenylalanine-threonine-tyrosine and phenylalanine-serine-tyrosine [22]. The in vivo inhibition of ACE seems supported, given that in our exploratory analysis reported herein, angiotensin II levels were significantly reduced at Week 8 relative to baseline levels in the RPC group. As angiotensin I and II levels were not assessed in participants in the placebo group, future studies are needed to determine whether RPC derived from Coldwater Shrimp is, indeed, associated with a reduction in angiotensin II levels. Also, whether the reduction in angiotensin II levels is a result of ACE inhibition also requires further investigation. As reported herein, 8 weeks after supplementation with RPC, there was a reduction from baseline in ACE activity; however, the reduction was not statistically significant.

In our study, treatment with RPC had a clinically relevant impact on lowering BP compared to placebo in the study participants, all of whom had mild to moderate hypertension at study entry. Overall, the primary outcomes—office SBP and daytime ambulatory SBP—were favourably affected by the consumption of RPC. With regard to office SBP, both the placebo and RPC groups experienced significant reductions from baseline at Weeks 2, 4, and 8, a finding that is not surprising, given the known placebo effect in hypertension studies [41]; however, between the two groups, the reductions in office SBP were significantly greater in the RPC group relative to the placebo group at Week 2 (by 2 mmHg; p=0.031) and Week 4 (by 2.2 mmHg; p=0.010) and trended toward a significant reduction at Week 8 (by 1.7 mmHg; p=0.087). Office BP measurements can be subject to observer bias and can be affected by temporary increases/decreases in BP due to clinic surroundings or an observer's presence, termed the ‘white-coat syndrome'; also, office BP measurements can be associated with blood pressure excursions that are situation-dependent. Automated BP measurement techniques, such as ABP monitors, overcome the limitations of office BP measurements [27]. With regard to ABP monitoring, participants receiving RPC treatment experienced significant improvements over those receiving a placebo in daytime SBP (at 4 and 8 weeks), night-time SBP (at 4 weeks), and 24-hour SBP (at 4 and 8 weeks). For other hypertension parameters, including ambulatory DBP, mean arterial pressure, and hypertension categorization, RPC outperformed placebo on all assessments. Importantly, the participants enrolled in this study were already in early progression towards developing hypertension or had mild or moderate hypertension. Thus, even modest reductions in BP are meaningful for this population; in fact, by the end of the study, the proportion of individuals who were categorized as being prehypertensive was significantly greater in the RPC group than in the placebo group, while the proportion of individuals categorized as having Stage 1 or 2 hypertension was significantly lower in the RPC group relative to the placebo group. Importantly, treatment with RPC was safe and well-tolerated.

## 5. Conclusions

Reported herein are the results of the first clinical study on the efficacy of RPC (a shrimp-derived protein hydrolysate) in reducing BP. Subjects recruited into this study had elevated BP (mild or moderate hypertension). RPC versus placebo significantly reduced BP in these subjects. From baseline to the end of the intervention period, RPC versus placebo caused a greater proportion of subjects to be shifted from hypertensive categories (i.e., Stage 1 or Stage 2 hypertension) into Normal/Prehypertensive categories. Findings from this study provide evidence that protein hydrolysates from Coldwater Shrimp can safely reduce BP for subjects with mild or moderate hypertension, possibly due to a reduction in angiotensin II levels. Further research is recommended to confirm the findings from this study (both on BP and mechanistic endpoints).

## Figures and Tables

**Table 1:** Demographic and lifestyle characteristics of the placebo and RPC groups in the ITT population (n=144).

Factor	Placebo (n = 72)	RPC (n=72)	p-value^a^
*Age (years)*			
Mean ± SD	54.9 ± 10.8	55.1 ± 9.8	0.923^b^
Median (Min – Max)	55 (33 – 72)	56.5 (32 – 73)	
*Location [n (*%*)]*			
North America	24 (33%)	23 (32%)	0.954
Czech Republic	31 (43%)	30 (42%)	
Germany	17 (24%)	19 (26%)	
*Gender [n (*%*)]*			
Female	28 (39%)	34 (47%)	0.400
Male	44 (61%)	38 (53%)	
*Alcohol Use [n (*%*)]*			
None	14 (19%)	13 (18%)	0.618
Occasionally	42 (58%)	40 (56%)	
Weekly	13 (18%)	18 (25%)	
Daily	3 (4%)	1 (1%)	
*Smoking Status [n (*%*)]*			
Current Smoker	1 (1%)	0 (0%)	1.000
Ex-Smoker	12 (17%)	13 (18%)	
Non-Smoker	59 (82%)	59 (82%)	
*Race [n (*%*)]*			
Black or African American	1 (1%)	3 (4%)	0.232
Central American	0 (0%)	2 (3%)	
Eastern European White	31 (43%)	32 (44%)	
Middle Eastern	0 (0%)	1 (1%)	
North American Indian/Aboriginal	0 (0%)	1 (1%)	
South American	0 (0%)	1 (1%)	
South Asian	5 (7%)	1 (1%)	
Western European White	35 (49%)	31 (43%)	
*Ethnicity [n (*%*)]*			
Hispanic or Latino	0 (0%)	4 (6%)	0.120
Not Hispanic or Latino	72 (100%)	68 (94%)	
*Status [n (*%*)]*			
Completed	69 (96%)	69 (96%)	1.000
Dropout/Withdrew	3 (4%)	3 (4%)	

ITT: intention-to-treat; Max: maximum; Min: minimum; n: number; RPC: Refined Peptide Concentrate; SD: standard deviation

^a^Between-group comparisons were made using Fisher's Exact Test, unless otherwise stated. p≤0.05 was considered statistically significant.

^b^Between-group comparison was made using the Independent Student's t-test.

**Table 2:** Baseline measurements for the placebo and RPC groups in the ITT population (n=144).

Variable	Placebo (n = 72)	RPC (n = 72)	p-value^a^
*SBP (mmHg)*			
Mean ± SD	147.3 ± 8.3	144.4 ± 6.5	0.019
Median (Min – Max)	146.5 (116.7 – 182.3)	144.0 (125.0 – 160.0)	
*DBP (mmHg)*			
Mean ± SD	88.1 ± 8.3	86.9 ± 6.7	0.329
Median (Min – Max)	89.7 (57.3 – 109.3)	86.0 (71.0 – 100.3)	
*Heart Rate (BPM)*			
Mean ± SD	71.3 ± 8.3	73.4 ± 8.7	0.139
Median (Min – Max)	73.3 (53.0 – 87.3)	72.0 (57.7 – 97.7)	
*Height (cm)*			
Mean ± SD	171.8 ± 9.7	171.0 ± 9.6	0.593
Median (Min – Max)	170.5 (149.2 – 195.0)	170.5 (150.0 – 188.0)	
*Weight (kg)*			
Mean ± SD	81.2 ± 13.4	81.2 ± 11.9	0.999
Median (Min – Max)	79.7 (60.1 – 119.0)	81.1 (61.4 – 116.9)	
*BMI (kg/m* ^*2*^)			
Mean ± SD	27.4 ± 3.1	27.8 ± 3.3	0.507
Median (Min – Max)	26.8 (22.8 – 34.9)	27.4 (20.9 – 34.8)	

BMI: body mass index; BPM: beats per minute; cm: centimeters; DBP: diastolic blood pressure; kg: kilograms; kg/m^2^: kilogram per square meter; Max: maximum; Min: minimum; mmHg: millimeter of mercury; n: number; RPC: Refined Peptide Concentrate; SBP: systolic blood pressure; SD: standard deviation

^a^Between-group comparisons were made using the Independent Student t-test. p≤0.05 was considered statistically significant.

**Table 3:** Changes in ambulatory SBP and DBP for all subjects in the ITT population during the 8-week supplementation period.

Ambulatory Measure	Group^a^	W 0	W 4	W 8	Change from W 0 to W 4^b^		Change from W 0 to W 8^b^	
					Within-group (p-value)	Between-group p-value	Within-group (p-value)	Between-group p-value
SBP (mmHg; mean ± SD)	*24-hour Ambulatory*							
	RPC	130.5 ± 10.3	130.2 ± 10.2	129.0 ± 9.6	-0.6 ± 11.5	0.015	-1.6 ± 9.0	0.006
					(p=0.684)		(p=0.138)	
	Placebo	132.4 ± 11.2	135.0 ± 11.5	134.4 ± 11.7	+2.4 ± 10.1		+1.8 ± 11.4	
					(p=0.052)		(p=0.200)	
	*Daytime Ambulatory*							
	RPC	136.1 ± 10.6	135.1 ± 11.1	134.0 ± 11.5	-1.3 ± 12.2	0.014	-2.2 ± 9.3	0.002
					(p=0.385)		(p=0.049)	
	Placebo	137.6 ± 12.7	140.4 ± 13.1	140.6 ± 12.9	+2.4 ± 12.4		+2.6 ± 13.9	
					(p=0.115)		(p=0.122)	
	*Night-time Ambulatory*							
	RPC	121.0 ± 11.8	120.4 ± 10.2	119.9 ± 9.5	-0.9 ± 14.8	0.007	-1.4 ± 13.9	0.166
					(p=0.613)		(p=0.408)	
	Placebo	124.0 ± 12.1	125.7 ± 12.5	123.1 ± 12.6	+2.0 ± 11.8		-1.0 ± 13.5	
					(p=0.165)		(p=0.551)	

DBP (mmHg; mean ± SD)	*24-hour Ambulatory*							
	RPC	80.1 ± 7.5	80.4 ± 8.3	80.1 ± 7.4	+0.1 ± 6.5	0.139	-0.1 ± 6.8	0.047
					(p=0.864)		(p=0.916)	
	Placebo	80.8 ± 8.2	82.4 ± 8.6	82.9 ± 8.7	+1.5 ± 8.0		+2.0 ± 9.9	
					(p=0.106)		(p=0.096)	
	*Daytime Ambulatory*							
	RPC	84.8 ± 8.1	84.3 ± 8.8	84.0 ± 8.6	-0.7 ± 6.4	0.036	-0.9 ± 7.2	0.004
					(p=0.367)		(p=0.294)	
	Placebo	85.1 ± 8.8	87.1 ± 9.5	88.2 ± 9.4	+1.8 ± 9.4		+2.9 ± 11.2	
					(p=0.115)		(p=0.034)	
	*Night-time Ambulatory*							
	RPC	72.8 ± 11.0	72.9 ± 8.6	73.2 ± 8.0	-0.2 ± 12.0	0.356	+0.1 ± 12.3	0.674
					(p=0.914)		(p=0.930)	
	Placebo	74.0 ± 10.3	74.1 ± 8.8	73.8 ± 9.4	+0.3 ± 9.4		-0.1 ± 11.8	
					(p=0.766)		(p=0.920)	

ANCOVA: analysis of covariance; DBP: diastolic blood pressure; ITT: Intention-to-treat; mmHg: millimeter of mercury; RPC: Refined Peptide Concentrate; SBP: systolic blood pressure; SD: standard deviation; W: week

^a^At W 0, either 1 or 2 subjects per group had ambulatory blood pressure recording errors and thus data from 70 or 71 subjects per group were used to generate ambulatory SBP and DBP values for W 0. Although ambulatory SBP and DBP data were available for all 72 subjects per group at W 4 and W 8, due to the aforementioned reason, data from 70 or 71 subjects per group were used to calculate changes from W 0 to W 4 and W 0 to W 8.

^b^Within-group comparisons of the changes from W 0 to W 4 and W 0 to W 8 were made using the paired Student t-test. Between-group comparisons of the change from W 0 to W 4 and W 0 to W 8 were made using ANCOVA with the value at baseline used as the covariate. p≤0.05 was considered statistically significant.

**Table 4:** Changes in office blood pressure for all subjects in the ITT population during the 8-week supplementation period.

Group	W 0	W 2	W 4	W 8	Change from W 0 to W 2^a^		Change from W 0 to W 4^a^		Change from W 0 to W 8^a^	
					Within-group (p-value)	Between-group p-value	Within-group (p-value)	Between-group p-value	Within-group (p-value)	Between-group p-value
*Office SBP* (mmHg; mean ± SD)										
RPC (n=72)	144.4 ± 6.5	139.9 ± 9.8	139.1 ± 8.5	137.7 ± 9.3	-4.4 ± 9.0	0.031	-5.3 ± 7.7	0.010	-6.7 ± 8.0	0.087
					(p<0.001)		(p<0.001)		(p<0.001)	
Placebo (n=72)	147.3 ± 8.3	144.9 ± 9.3	144.2 ± 9.4	142.3 ± 11.4	-2.4 ± 8.4		-3.1 ± 9.3		-5.0 ± 10.8	
					(p=0.017)		(p=0.007)		(p<0.001)	
*Office DBP* (mmHg; mean ± SD)										
RPC (n=72)	86.9 ± 6.7	85.2 ± 7.2	85.0 ± 7.7	85.2 ± 7.2	-1.7 ± 5.5	0.515	-1.9 ± 7.0	0.081	-1.7 ± 6.5	0.137
					(p=0.010)		(p=0.027)		(p=0.033)	
Placebo (n=72)	88.1 ± 8.3	86.6 ± 7.5	87.5 ± 7.0	87.5 ± 8.0	-1.5 ± 6.7		-0.6 ± 6.8		-0.6 ± 7.7	
					(p=0.055)		(p=0.422)		(p=0.492)	

ANCOVA: analysis of covariance; DBP: diastolic blood pressure; ITT: intention-to-treat; mmHg: millimeter of mercury; RPC: Refined Peptide Concentrate; SBP: systolic blood pressure; SD: standard deviation; W: week

^a^Within-group comparisons of the changes from W 0 to W 2, W 0 to W 4, and W 0 to W 8 were made using the paired Student t-test. Between-group comparisons of the changes from W 0 to W 2, W 0 to W 4, and W 0 to W 8 were made using ANCOVA with the value at baseline used as the covariate. p≤0.05 was considered statistically significant.

**Table 5:** Results from Exploratory Analyses for Participants in the RPC Group.

	Oxidized LDL (U/L)	ACE (ng/mL)	Renin (pg/mL)	Angiotensin I (pg/mL)	Angiotensin II (pg/mL)	Aldosterone (pg/mL)
W 0						
Mean ± SD	111.4 ± 27.8	151 ± 59	645 ± 340	1,343 ± 850	0.309 ± 0.148	375 ± 142
Median (Min – Max)	116.6 (43.4 – 150.2)	142 (51 – 337)	584 (84 – 2,007)	1,045 (29 – 4,311)	0.275 (0.197 – 0.953)	364 (103 – 726)
W 8						
Mean ± SD	110.9 ± 21.2	138 ± 46	640 ± 382	1,383 ± 1,134	0.283 ± 0.148	366 ± 128
Median (Min – Max)	113 (52.3 – 141.8)	130 (55 – 289)	589 (201 – 2,679)	1,042 (42 – 6,155)	0.217 (0.186 – 0.968)	370 (105 – 722)
Change from W 0 to W 8						
Mean ± SD	-1 ± 33	-12 ± 54	-5 ± 327	39 ± 796	-0.026 ± 0.060	-9 ± 108
Median (Min – Max)	-6 (-64 – 95)	-5 (-141 – 89)	-26 (-1,201 – 1,203)	-2 (-2,738 – 3,428)	-0.01 (-0.176 – 0.101)	-8 (-264 – 393)
P-value	p=0.594^*α*^	p=0.176*∗*	p=0.895*∗*	p=0.532*∗*	p<0.001*∗*	p=0.596^¤^

ACE: angiotensin converting enzyme; LDL: low-density lipoprotein; Max: maximum; Min: minimum; mL: milliliter; ng: nanogram; pg: picogram; SD: standard deviation; U/L: International units per liter; W: week.

Within-group comparisons were made using the paired Student t-test.

^*∗*^Logarithmic transformation required to achieve normality.

^*α*^Squared transformation required to achieve normality.

^¤^Square root transformation required to achieve normality.

Probability values P≤0.05 are statistically significant.

## Data Availability

Requests for access to individual subject data may be made to Marealis AS; please send an email to andreas@marealis.no.
